# Subducting seamounts control interplate coupling and seismic rupture in the 2014 Iquique earthquake area

**DOI:** 10.1038/ncomms9267

**Published:** 2015-09-30

**Authors:** Jacob Geersen, César R. Ranero, Udo Barckhausen, Christian Reichert

**Affiliations:** 1 GEOMAR Helmholtz Centre for Ocean Research Kiel, Wischhofstrasse 1-3, 24148 Kiel, Germany; 2 Barcelona Center for Subsurface Imaging, Instituto de Ciencias del Mar, ICREA at CSIC, Passeig Marítim de la Barceloneta 37-49, 08003 Barcelona, Spain; 3 Bundesanstalt für Geowissenschaften und Rohstoffe (BGR), Stilleweg 2, 30655 Hannover, Germany

## Abstract

To date, the parameters that determine the rupture area of great subduction zone earthquakes remain contentious. On 1 April 2014, the Mw 8.1 Iquique earthquake ruptured a portion of the well-recognized northern Chile seismic gap but left large highly coupled areas un-ruptured. Marine seismic reflection and swath bathymetric data indicate that structural variations in the subducting Nazca Plate control regional-scale plate-coupling variations, and the limited extent of the 2014 earthquake. Several under-thrusting seamounts correlate to the southward and up-dip arrest of seismic rupture during the 2014 Iquique earthquake, thus supporting a causal link. By fracturing of the overriding plate, the subducting seamounts are likely further responsible for reduced plate-coupling in the shallow subduction zone and in a lowly coupled region around 20.5°S. Our data support that structural variations in the lower plate influence coupling and seismic rupture offshore Northern Chile, whereas the structure of the upper plate plays a minor role.

## Abstract (web summary)

On 1 April 2014 the Mw 8.1 Iquique earthquake seemed to close the well-recognized northern Chile seismic gap, producing only a small rupture. Here, the authors present seismic reflection and multibeam bathymetry data from the area suggesting that seamount subduction played a role in halting the rupture.

## Graphical abstract

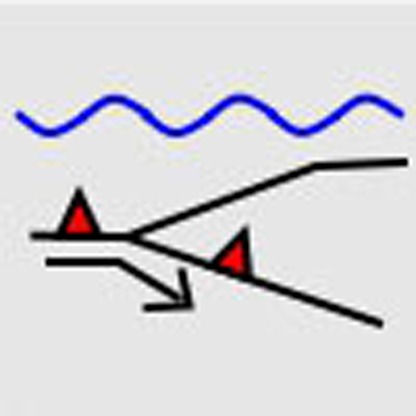

The Chilean subduction zone can be divided into different seismo-tectonic segments that are known to have repeatedly ruptured over the last centuries during great and giant megathrust earthquakes every ∼100–150 years^1^. This pattern of recurring earthquakes defines the northern Chile seismic gap (∼18°S–24°S; Fig. 1a) with a last great event about 137 years ago (∼Mw 8.6 Iquique earthquake in 1877)^1^. The anticipation of an impending great earthquake has motivated numerous international field campaigns onshore, making the area one of the best-studied seismic gaps worldwide. Along its ∼550 km, the seismic gap is characterized by a heterogeneous plate-coupling distribution (Fig. 1b)^2^,^3^,^4^. South of 21°S a highly coupled patch is presented with moderate to high coupling (>0.5) extending into the shallow-most part of the subduction zone. In contrast, in the central part of the seismic gap (19–20.5°S) high coupling is only met in a small patch (>60 km landward from the deformation front) and decreases to values <0.5 towards the trench. The two patches are separated by a lowly coupled region around 20.5°S. North of 19°S coupling is less well constrained due to the large distance of the coastline^2^,^3^,^4^. Similar to most subduction zones, the vast majority of stress accumulation across the North Chilean plate boundary occurs offshore, where plate coupling is highest. Understanding deformation patterns offshore is thus important for studying seismogenesis at the margin.

In January 2014, a series of earthquake swarms were followed by the 1 April 2014 Mw. 8.1 Iquique earthquake that, together with a Mw. 7.6 aftershock 2 days later, ruptured the moderately coupled central part of the seismic gap^2^,^5^,^6^,^7^,^8^,^9^ (Fig. 1b). Different slip models have been published for the earthquake deviating in the absolute amplitude of peak slip (between ∼4 and ∼7 m)^2^,^5^,^6^,^7^,^8^,^9^. However, all slip models comply that earthquake rupture extended only over a ∼150-km-long stretch (between ∼19 and 20.5°S) and did not migrate farther along-strike into the highly coupled southern part of the seismic gap. The slip models also agree on the fact that peak slip occurred down-dip of the hypocentre and that earthquake rupture diminished towards the shallow-most up-dip part of the subduction zone.

Here, we present an unpublished compilation map of swath bathymetry of the seafloor and marine multichannel seismic reflection images of the overriding plate and plate-boundary fault zone of the northern Chile seismic gap. The new data show multiple large seamounts on the oceanic plate seaward of the deformation and along the plate interface under the marine forearc. Those subducting seamounts likely exert a primary control on regional-scale plate-coupling variations and on the limited up-dip and southward extent of seismic rupture during the 2014 Iquique earthquake.

## Results

### Oceanic plate structure seaward of the deformation front

**Figure 1:**
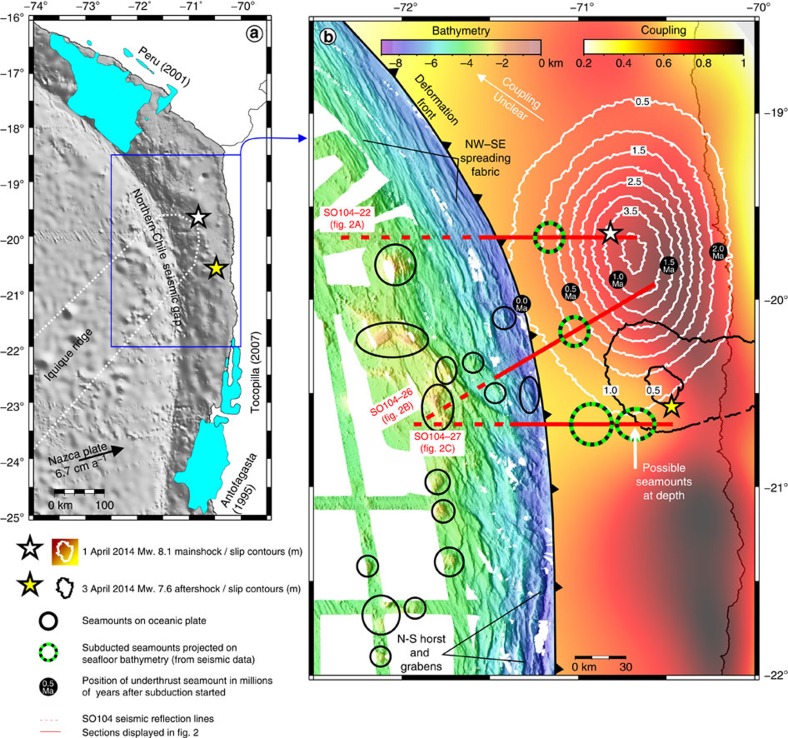
Map of northern Chile and southern Peru. (**a**) Seafloor bathymetry offshore northern Chile and southern Peru (data from GEBCO_08 Grid; version 20091120) including the slip areas of the 1995 Antofagasta, 2001 Peru and 2007 Tocopilla earthquakes^22^,^23^,^24^. The black arrow indicates the average annual movement of the Nazca Plate with respect to South America^25^. (**b**) Compilation of swath bathymetry (seaward of the deformation front) and plate-coupling^2^ (landward of the deformation front) for the northern Chile seismic gap. Slip contours of the 1 April 2014 Mw 8.1 Iquique earthquake and the 3 April 2014 Mw 7.6 aftershock from ref. 2 The oceanic plate off the highly coupled southern segment of the seismic gap is characterized by large N–S trending horst and grabens. Seaward of the moderately coupled central part that ruptured during the 2014 Iquique earthquake individual seamounts of the Iquique Ridge alter the morphologic signature of the oceanic plate. Around 19°S the structure of the oceanic plate changes again, from a complex three-dimensional setting (seamounts, horst and grabens, spreading fabric) in the south to a more simple setting (spreading fabric only) farther north.

The moderately coupled central part of the seismic gap^2^,^3^,^4^ that ruptured during the 2014 Iquique earthquake matches the width of the Iquique Ridge interpreted to be subducted under the marine forearc (Fig. 1)^4^,^10^. The ridge, however, does not form a spatially continuous prominent swell in the seafloor of the incoming plate. Instead, the oceanic plate close to the subduction zone is characterized by a subdued topography, slightly shallower (yellowish colours) than the surrounding seafloor (green colours), that is overprinted by multiple (>10) conical seamounts in the limited area with swath bathymetric coverage (Fig. 1b).

### Deformation of the plate boundary and overriding plate

**Figure 2:**
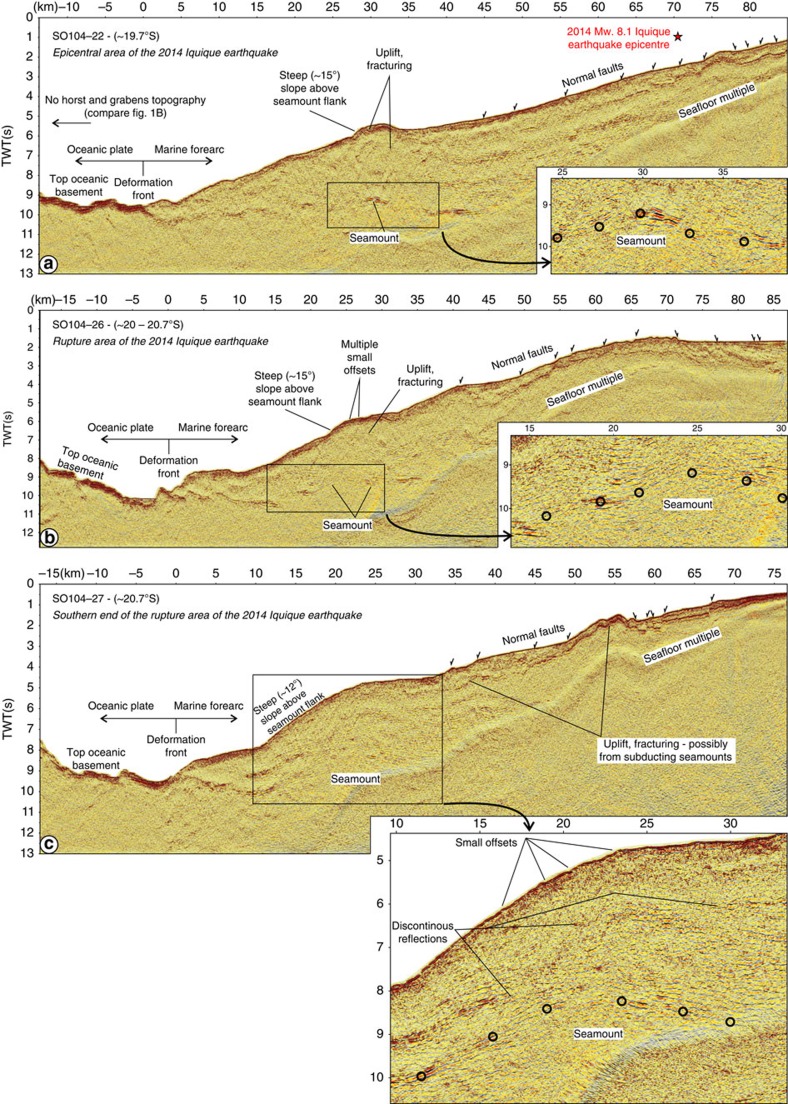
Seismic reflection data from the northern Chile seismic gap. (**a**) Time section of seismic reflection line SO104-22. The 1 April 2014 Mw. 8.1 Iquique earthquake epicentre is indicated by the red star. Seaward of the deformation front the top of the oceanic basement lies exposed at the seafloor, indicating the absence of trench sediments. A spatially fairly continuous high-amplitude top oceanic basement reflection can be traced under the marine forearc to kilometre 45 where seismic resolution diminishes due to the presence of the seafloor multiple. Around kilometre 30 an upward bulge (∼2.3 km (1 s TWT) high, ∼12 km wide) in the subducting oceanic basement indicates the presence of a subducting seamount. The morphological response of the upper plate is expressed by local steepening of the continental slope above the seamount (to ∼15°) as well as localized uplift and fracturing. The upper continental slope is depicted by landward and seaward dipping normal faults. (**b**) Time section of seismic reflection line SO104-26. Similar to the northern line a subducting seamount, ∼2.3 km (1 s TWT) high and ∼15 km wide, is located under the marine forearc resulting in extensive localized deformation of the upper plate. The upper continental slope is depicted by a series of landward and seaward dipping normal faults. (**c**) Time section of seismic reflection line SO104-27. A large (∼4 km (1.8 s TWT) high, ∼20 km wide) subducting seamount is located under the marine forearc between kilometres ∼10 and 30. In addition to steepening of the continental slope (to ∼12°), multiple small offsets of the seafloor and shallow strata above discontinuous deeper reflections indicate extensive deformation of the upper plate above the seamount. Farther landward, between kilometres 33–45 and 48–58 localized uplift and fracturing of upper-plate strata indicate the possible presence of two additional seamounts at depth. The seamounts possibly prevented seismic rupture during the 2014 Iquique earthquake from migrating southwards into highly coupled southern part of the northern Chile seismic gap. Landward and seaward dipping normal faults characterize the upper continental slope.

However, we find that seamounts are not only located seaward of the deformation front, but also on the downgoing plate under the marine forearc (Fig. 2). About 20 km landward of the deformation front, at the southern end of the area that ruptured during the 2014 Iquique earthquake, a large seamount (1.8 s two-way-traveltime (TWT) high and ∼20 km wide) is subducting under the marine forearc (Fig. 2c; also compare black–green circles in Fig. 1b for projection of subducting seamounts on seafloor bathymetry). With an average seismic velocity of 4.5 km s^−1^, as inferred from wide-angle seismic data farther to the south^11^,^12^, the relief of the seamount corresponds to an absolute height of ∼4 km. The under-thrusting of the seamount results in manifold deformation of the upper plate (Fig. 2c). Above its seaward flank the continental slope steepens to ∼12°, indicating large-scale failure of the entire upper plate in the wake of the subducting seamount, a deformation pattern observed elsewhere in continental margins affected by seamount subduction^13^. Multiple small offsets of the seafloor and shallow strata above discontinuous deeper reflections indicate extensive faulting cutting across much of the upper plate. Down-dip of the seamount, the plate boundary is not well imaged due to the presence of the seafloor multiple. However, localized uplift and fracturing of the seafloor and upper-plate strata between kilometres 33–45 and 48–58 suggest the possible presence of two additional seamounts or similar extensive relief on the subducting plate at depth. Two other seamounts that induce a similar deformation pattern in the upper plate are imaged in seismic lines SO104-22 (Fig. 2a, 25–37 km) and SO104-26 (Fig. 2b, 16–31 km). Both seamounts are about ∼2.3 km (∼1 s TWT) high, and ∼12–15 km wide.

### Temporal and spatial extent of seamount subduction

The sparse seismic lines do not provide information on how far and widespread seamounts have been subducted, but kinematic reconstructions^10^ support that initial ridge subduction occurred over the last 2 Ma. The black dots in Fig. 1b illustrate the down-dip extent of a subducting seamount for a 2-Ma time period using a plate-dip of 25° and a convergence rate of 6.7 cm a^−1^. This shows that the seamounts imaged by the seismic data started to subduct over the last ∼1 Ma, which is in agreement with the kinematic reconstructions. However, from the seismic and swath bathymetric data, we cannot infer on the presence/or absence of additional seamounts in the deeper part of the subduction zone or between the seismic lines.

## Discussion

The multiple seismically imaged subducting seamounts (Fig. 2) indicate that the moderately coupled central part of the northern Chile seismic gap, which ruptured during the 2014 Iquique earthquake, and the lowly coupled area around 20.5°S have a distinct tectonic evolution. Here, the physical state of the plate-boundary fault zone is widely affected by the presence of excess relief on the subducting plate. Subducted seamounts and, more generally, variations in subducting seafloor roughness are believed to influence the characteristics of plate-coupling and seismic rupture at different plate boundaries^14^,^15^,^16^,^17^. Wang and Bilek^17^ argue that a subducting seamount causes pervasive fracturing around the initial plate boundary, therewith creating favourable conditions for aseismic creep and small earthquakes and unfavourable conditions for large earthquake rupture to propagate across that area. The seismic reflection lines image the plate-boundary fault zone as a group of reflections, possibly formed by several sub-parallel surfaces containing fluids. From this we cannot identify the main decollement, which may be only a few centimetres thick. However, extensive localized deformation in the upper plate above and around the subducting seamounts, as imaged by the seismic data, supports the interpretation that in the area of the 2014 Iquique earthquake the entire overriding plate, including the plate-boundary fault zone, is widely fractured and faulted by the deformation caused by the subducting seamounts.

The deformation associated with 2–4-km-tall under-thrusting seamounts (Fig. 2), of the extension of the Iquique Ridge now located under the marine forearc, may cause the conditions that prevent the long-term accumulation of elastic energy in the shallow-most up-dip part of the subduction zone as well as in the lowly coupled region around 20.5°S (Fig. 1b). Furthermore, the seamounts imaged at the southern end of the rupture area of the 2014 Iquique earthquake that extend to the depth of peak slip (around 20.5°S; SO104-27, Fig. 2c) possibly physically prevented seismic rupture during the 2014 Iquique earthquake from migrating southwards, into the highly coupled southern area of the northern Chile seismic gap. Similar seamounts imaged at the shallow plate interface (Fig. 2a,b) might have further prevented seismic rupture from propagating into the shallow-most up-dip part of the subduction zone.

South of 21°S the oceanic plate facing the highly coupled southern segment of the northern Chile seismic gap lacks large seamounts. Instead, oceanic plate morphology at the trench is controlled by prominent (0.5–1.5 km vertical displacement) horst and grabens that result from bend-faulting of the incoming plate (Fig. 1b). A similar subducting plate morphology is observed offshore large areas of northern Japan, where seismic rupture during the giant 2011 Mw 9.0 Tohoku-Oki earthquake extended up-dip to the trench triggering the large tsunami^18^,^19^. In contrast, north of 19°S where plate-coupling is not well constrained, neither large seamounts nor bending-related horst and grabens are observed seaward of the deformation front (Fig. 1b). Instead, comparatively smaller bending-related half grabens are formed along the trench by reactivation of NW–SE trending spreading fabric formed at the palaeo-spreading centre^20^,^21^ (Fig. 1b).

Our data support that the change to a complex three-dimensional plate interface, which is affected by the subduction of large seamounts in the moderately coupled central part (19–20.5°S) of the northern Chile seismic gap, possibly exerts a first-order control on plate-coupling and seismic rupture. During the 2014 Iquique earthquake, the subducted seamounts may have physically limited along-slope and up-dip migration of seismic rupture. Furthermore, the deformation associated with the under-thrusting seamounts possibly creates favourable conditions for aseismic creep through extensive fracturing around the initial plate-boundary fault zone, therewith favouring the overall reduced coupling rates in the shallow-most up-dip part of the subduction zone as well as in the lowly coupled region around 20.5°S. Although our results support a segmentation of the seismic gap, a more cumulative earthquake record is necessary to understand how smaller events such as the 2014 Mw. 8.1 Iquique earthquake interact with bigger events that historically have ruptured the entire segment.

## Methods

### Data Collection

Seismic reflection lines off Northern Chile were collected in 1995 by the Bundesanstalt fuer Geowissenschaften und Rohstoffe during R/V *Sonne* cruise SO104 in the framework of the Crustal investigations off- and onshore Nazca/Central Andes (CINCA) project. Seismic data were collected with a 3-km-long digital streamer with 25-m-long active channels, and a ∼3,124-cubic-inch (51.2 l), well-tuned source with two airgun strings shot every 50 m.

### Data Processing

Processing of seismic data was conducted with the Claritas software package. After data editing, the traces were cleaned with a pre-stack wavelet-shaping statistical deconvolution. Later, shot point interpolation and offset regularization was done for two-dimensional filtering. Semblance-type velocity analyses were interpreted every 5 km. The water-layer multiple was attenuated with parabolic radon filtering on super gathers after normal-moveout correction with stacking velocities. A pre-stack time migration was done on receiver gathers with time and space varying velocities based on the velocity analysis. After pre-stack time migration, new velocity analyses were interpreted for common-mid-point stacking. After stacking, the data were post-stack time migrated using a finite difference algorithm, with velocities following the geological structure. For the shallow geology, seismic velocities were based on velocity analysis, whereas deeper in the section, seismic velocities were based on wide-angle seismic profiles from the margin^11^,^12^. After post-stack time migration, the data were bandpass filtered with time- and space-varying frequencies, based on geological structure. For display purposes, the data are presented with an automatic gain control normalization based on the mean amplitude within a sliding window of 1,000 ms.

## Additional information

**How to cite this article:** Geersen, J. *et al*. Subducting seamounts control interplate coupling and seismic rupture in the 2014 Iquique earthquake area. *Nat. Commun.* 6:8267 doi: 10.1038/ncomms9267 (2015).
